# Intubation of the Larynx for Croup

**Published:** 1886-12

**Authors:** Henry D. Ingraham

**Affiliations:** Professor of Diseases of Women and Children, Medical Department of Niagara University; 405 Franklin Street


### INTUBATION OF THE LARYNX FOR CROUP.

By HENRY D. INGRAHAM, M. D.

Professor of Diseases of Women and Children, Medical Department of Niagara University.

In the October number of this JOURNAL I reported two cases of intubation of the larynx for diphtheritic croup. Both patients were then wearing the tubes, and in Case I. the symptoms were favorable for the recovery of the child. This was at the end of the third day. As is usual in these cases the child was quite thirsty; was not satisfied with the ice, which was given freely, and a teaspoonful of water occasionally.

The patient begged for water, and her mother gave her a tumblerful, all of which she drank, although strict orders were given to allow but a teaspoonful at a time, and that was to be given with the patient lying on her side.

Immediately the child had a severe paroxysm of coughing and in twenty minutes was dead. No doubt she was suffocated or drowned by the passage of considerable of the water through the tube into the lungs, as when last seen by Dr. Banta and myself, a few hours before, she appeared to be much better than at any previous time.

In Case II. the child did not experience as much relief at any time as in Case I., because at the time of the introduction of the tube the disease had involved the bronchi quite extensively. This child died the third day after the introduction of the tube.

Tuesday, Nov. 2d.—I was called by Drs. Hartwig and Krug, to see a child four years old, who had been suffering from membraneous croup for nine days. And although it was not expected to be of much benefit, the child being too near its end, yet the tube was inserted about five o'clock P. M. Considerable relief was afforded the child at first, but in a short time the breathing was as bad as it had been before the introduction of the tube, and at two o'clock in the morning the child died.

These three cases are all that I have seen, and although they all proved fatal, yet I believe in the utility of intubation. The relief given the little sufferers was sufficient to justify the proceedure, even though they did not recover. In Case I. the relief was so marked that every one who saw the patient was thankful the tube had been introduced. The child said to us, soon after the introduction of the tube, "you are good doctors to make me feel so much better."

These cases illustrate the necessity of great care and attention in the subsequent treatment after intubation for diphtheritic or membraneous croup. If the relief be as marked, as in some cases, it is with difficulty that you can convince the friends that the patient is still suffering from a very grave disease, and that it is necessary to do everything possible to aid the patient's recovery.

Another necessity to success is the early introduction of the tube. If we wait until the child is almost moribund there is nothing gained, except more or less temporary relief, which, in many cases, is sufficient to justify its use even at a late period in the disease. In the above cases wearing the tube produced no irritation nor inconvenience to the patient.

The objections to the use of the tube are:

First—The difficulty of introduction, which, in some cases, is great, yet it is not sufficient to prevent all being done to relieve the patient that is possible, especially as this is an objection on the part of the physician and not an objection upon the part of the patient or friends.

Second—The thirst of the patient and its inability to swallow liquids. With an intelligent nurse, who will carry out your directions, a child can swallow a teaspoonful of liquid at a time, when lying on the side, without any of it passing through the tube to the lungs, and this small amount, with what ice can be given, is sufficient to quench thirst, or, at least, to answer the requirements of the system. I know of no other objections to the use of the tube. The percentage of recoveries following intubation is reported greater than that following tracheotomy, and certainly the objections to it are much less.

When we can have an intelligent nurse, one who will carefully attend to the administration or non-administration of food and drinks and conscientiously carry out our instructions, I believe the recoveries from intubation will be much greater than at present reported, and the operation come into more frequent use.

405 Franklin Street.

